# Prevalence of myopia in Indian school children: Meta-analysis of last four decades

**DOI:** 10.1371/journal.pone.0240750

**Published:** 2020-10-19

**Authors:** Divya Agarwal, Rohit Saxena, Vivek Gupta, Kalaivani Mani, Rebika Dhiman, Amit Bhardawaj, Praveen Vashist

**Affiliations:** 1 Department of Ophthalmology, Dr. Rajendra Prasad Centre for Ophthalmic Sciences, All India Institute of Medical Sciences, New Delhi, India; 2 Department of Community Ophthalmology, Dr. Rajendra Prasad Centre for Ophthalmic Sciences, All India Institute of Medical Sciences, New Delhi, India; 3 Department of Biostatistics, All India Institute of Medical Sciences, New Delhi, India; Institute of Economic Growth, INDIA

## Abstract

**Background:**

India is the second most populated country in the world with 41% of the population (492 million) under 18 years of age. While numerous studies have shown an increasing prevalence of myopia worldwide, there continues to be uncertainty about the magnitude of myopia in Indian school going population.

**Design:**

Systematic review and meta-analysis.

**Methods:**

We systematically identified published literature of last four decades from 1980 to March 2020 and assessed them for methodological quality. Data were gathered into 5-year age groups from 5–15, in urban or rural populations, and standardized to definition of myopia as refractive error ≥ -0.50 dioptre. Random effects meta-analysis was done.

**Results:**

We included data from 59 quality assessed studies, covering nearly 1,66,000 urban and 1,20,000 rural children. The overall crude prevalence of myopia over last four decades is 7.5% (95% CI, 6.5–8.5%) in 5-15-year age group. The prevalence of myopia is 8.5% (95% CI, 7.1–9.9%) in urban and 6.1% (95% CI, 4.5–7.7%) in rural children, with highest prevalence in urban 11-15-year age group [15.0% in last decade]. A significant increment in prevalence is noted in the last decade in rural children from 4.6% to 6.8%, reflecting changing rural environment.

**Conclusion:**

Myopia is an emerging public health problem in both urban and rural school going adolescents in India requiring urgent efforts.

## Introduction

Rising prevalence of myopia is a major challenge worldwide, giving rise to an epidemic in certain regions. It is the most common refractive error and an important cause of ocular morbidity especially affecting school going children and young adults. Uncorrected myopia has huge social, economic, psychological and developmental implications [1]. Various studies in the literature have predicted dramatic rise of myopia in the coming years causing a great concern among stakeholders and is projected to affect 50% of world population by 2050 [2]. There is a large regional variation in the myopia prevalence with the dominance of East Asian countries that report a far greater prevalence as compared to other countries [2, 3].

India is the second most populated country in the world, with around 41% of its population (492 million) being less than 18 year age group [4]. This young population is an important asset for development of the country and their challenges must be addressed in time. While rising myopia is a cause of concern in most of the countries, it is not given due importance in India due to lack of adequate nationwide prevalence data and prospective studies comparing the trend of myopia over decades [5]. Due to this, the representation of India is poor in studies predicting global trends of myopia [6]. Previous studies by the authors have reported a prevalence of myopia of only 13.1% among school going children in north India with an annual incidence of 3.4% [7, 8]. However, due to the large regional differences in culture, habits, socioeconomic status, educational levels and urbanisation, there continues to be an uncertainty about the exact magnitude of myopia burden in Indian school going children and its trend over time. The study was undertaken to fill up this lacuna which can help in understanding the prevalence of myopia, regional variations and prediction of trend, using all the published literature of the last four decades from India.

## Methods

The study followed the Preferred Reporting Items for Systematic Reviews and Meta-Analyses (PRISMA) guidelines for the purpose of this review.

### Search strategy

We performed a systematic search and review of the prevalence of myopia in India using published data of the last four decades. We searched PubMed, Medline, Embase, OVID, Web of Science, CINAHL and Cochrane library databases from 1^st^ January 1980 to 31^st^ March, 2020. Many research articles from India are not available in PubMed search. Thus, we also searched other indexing systems- Index Copernicus and Google Scholar to make our search more inclusive. The search was restricted to all the online available articles mentioning prevalence of myopia in any region of India and published till March 2020. We searched these databases using the following MeSH (Medical Subject Heading) terms and keywords: myopia AND prevalence AND India and refractive error AND prevalence AND India. Broad search strategy also used terms related to epidemiology like epidemiology, incidence, rates, proportion and prevalence, terms related to disease (including medical subject headings search using exp refractive error*, exp myopia* and keyword search using the terms refractive error, myopia and shortsight*) and terms related to population (including medical subject headings search using exp India* and keyword search using the word India). We also identified and included relevant studies by manually searching the reference lists of eligible studies. Further details about search strategy are available in S1 File.

### Definitions, inclusion and exclusion criteria and quality assessment

We conducted an initial broad search focusing on all studies that estimated the prevalence and/or incidence of refractive errors and/ or myopia among all age groups from any region in India. Later, the search was restricted to age group of 5–15 years for this systematic review. Prevalence was defined as the number of individuals in a population that have myopia at a given point of time divided by those at risk. Myopia was defined as spherical equivalent of -0.5 Dioptre or worse [2, 7–9]. This standard definition was applied to most of the studies to shortlist them for data abstraction. We covered both urban and rural settings, making our search more representative as majority of Indian population resides in rural villages. Cross-sectional studies including population-based as well as school-based studies were included. Qualitative studies, review articles, articles published in languages other than English and articles which did not have relevant information available online were excluded. A data extraction form was later developed to include all the studies which met our inclusion criteria. Various study characteristics like study design, study population, study location/ region, demographic details (age, gender), screening tools, case definitions used and epidemiological data were compiled from the above studies. We extracted separate urban and rural myopia prevalence rates and gender-based rates, wherever possible. The data was combined and later stratified in each 5-year age groups- 5–10; 11–15 years wherever possible. A detailed uniform methodological quality assessment of each of the included study was done by three independent observers using the critical appraisal checklist developed for prevalence studies by Hoy et al. (2012) [10]. Those studies which obtained aggregate score more than six were labelled as ‘high risk’ studies. Those studies which obtained aggregate score less than four and between four-six were labelled as ‘low risk’ and ‘moderate risk’ studies, respectively. Final score was decided based on consensus among the three observers.

### Statistical methods

Meta-analysis was carried out using Stata 12.0 (StataCorp LP, Texas, USA). The random effects model using DerSimonian and Laird method was used to calculate pooled effect sizes and its 95% confidence interval (CI) limit [11]. Forest plots were generated displaying prevalence of myopia with corresponding 95% CI. The variation in the magnitude of the effect was examined and heterogeneity was quantified using I^2^ statistic. The funnel plot was used to detect potential reporting bias and small/large study effects and Egger method was used to assess asymmetry.

Studies which were categorised as ‘high risk’ based on assessment of methodological quality described above, were excluded from the final analysis. All studies (low, moderate and high risk) were included in a sensitivity analysis. Urban and rural data was analysed separately. The studies which represented both urban and rural population were later subdivided into separate datasets based on study settings (urban or rural) for detailed analysis. Rural-urban and time-stratified estimates of prevalence of myopia across included studies were obtained. For time stratified estimates, year of publication of study was taken for subgroup analysis unless study period was mentioned in the study. Decadal variation was assessed by subgroup analysis of 2009–2019 studies with those of previous decades. Results of rural were compared with urban studies, and studies conducted during 2009–2019 were compared with older studies, by computing z-scores. A sub-group separate analysis for children aged 11–15 years was also done for urban and rural studies. No such analysis can be carried out in 5-10-year age group due to limited number of available studies.

## Results

Using the above described search strategy, 469 potentially relevant titles/ abstracts were identified, 165 relevant articles were assessed for eligibility, and 77 studies were found to be eligible [12–87]. The detailed quality assessment of eligible studies is reported in S1 Table. 18 studies were found to be “high risk”. Thus, 59 studies were included in the main analysis, while data from all 77 eligible studies were included in sensitivity analysis. The summary of review strategy is presented in S1 Fig.

Out of 59 studies included in main analysis, 37 showed representation of only urban data, 12 showed only rural data and 10 studies showed both urban and rural data. Region wise representation of studies is as follows: North India (12), North East India (4), Central India (6), East India (7), West India (8) and South India (22). All studies were cross sectional in nature. Most of the studies were school-based, with only 4 being population-based. Most of the studies were conducted in the last decade (2009–2019) with only few studies being available before 2009. Gender-based data was available only in few studies, precluding a gender stratified analysis. Overall, the review included around 1,66,000 urban school going children and 1,20,000 rural children over the last 4 decades. The studies were stratified into urban and rural settings and separate analysis was done for the same. Those studies which represented both urban and rural study settings were subdivided into separate datasets as urban or rural subset depending on study setting and data availability. Nine additional datasets were created to represent nine studies where data was available separately for urban and rural setting. The total number of datasets included in analysis were 68 (59 original studies + 9 additional datasets). The details of studies which were included for final statistical analysis are presented in Table 1, along with their study coverage area (urban/ rural/ both). One study did not give rural-urban data separately and was excluded from rural-urban sub-group analysis [22].

**Table 1:** Characteristics of various studies that reported data on prevalence of myopia in Indian school-age children and are included in the final meta-analysis.

S No.	First Author (Year of Publication) [Citation]	Study Place	Region (India)	Coverage	Age Group (years)	Coverage	Cycloplegic Refraction	Total Sample Size (Subdivision when both urban and rural data separately available)	No. of myopic cases (Subdivision when both urban and rural data separately available)	Prevalence of myopia (%)	Overall Quality Assessment Score**
1	Ahmed (2008) [12]	Srinagar	North	School	6–22	Urban	Yes	4360	207	4.74	Low Risk
2	Bansal (2012) [13]	Kolar	South	School	6–16	Urban	Yes	2680	307	11.5	Moderate risk
3	Aroor (2014) [14]	Surathkal	South	School	4–16	Urban	Yes	755	102	13.5	Moderate risk
4	Ande (2015) [15]	Guntur	South	School	10–15	Rural	Yes	3174	148	4.66	Low Risk
5	Mondal (2014) [16]	Kolkata	East	School	8–17	Urban	Yes	1649	128	7.76	Low Risk
6	Gupta (2012) [17]	Shimla	North	School	5–15	Urban	Yes	2000	48	2.4	Low Risk
7	Datta (1983) [18]	Kolkata	East	School	5–13	Urban	Yes	24007	216	0.89	Moderate risk
8	Batra (2007) [19]	Ludhiana	North	School	5–15	Urban, Rural	Yes	19610 (Urban- 11185, Rural- 8425)	1366 (Urban-1115, rural-251)	6.97	Low Risk
9	Chandra (1982) [20]	Prayagraj	Central	School	8–16	Urban	Yes	8600	1430	16.43	Moderate risk
10	Chatterjee (2014) [21]	Kolkata	East	School	5–14	Urban	Yes	16597	960	5.78	Low Risk
11	Dandona (2002a) [22]	Hyderabad, West Godavari, Adilabad, Mahbubnagar	South	Population	0–15	Urban, Rural	Yes	1810 (5–15 yr age)	66	3.6	Low Risk
12	Dandona (2002b) [23]	Mahbubnagar	South	Population	7–15	Rural	Yes	4074	163	4.1	Low Risk
13	Das (2007) [24]	Kolkata	East	School	5–10	Urban	NA*	2317	325	14.02	Moderate risk
14	Agrawal (2018) [25]	Raipur	Central	School	5–15	Urban, Rural	NA	1557 (urban- 836, rural- 721)	50 (urban- 36, rural- 14)	3.21	Low Risk
15	Dhanya (2016) [26]	Bangalore	South	School	5–15	Urban	Yes	958	45	4.7	Moderate risk
16	Ganapathi (2017) [27]	Salem	South	School	10–17	Urban	NA	828	98	11.8	Moderate risk
17	Ghosh (2012) [28]	Kolkata	East	School	6–14	Urban	Yes	2732	307	11.23	Low Risk
18	Singh (2013) [29]	Bhopal	Central	School	5–15	Urban, Rural	Yes	18500 (Urban-7955, Rural-10545)	1313 (Urban-299, rural-1014)	7.09	Moderate risk
19	Krishnamurthy (2014) [30]	Mysore	South	School	5–15	Urban, Rural	Yes	1123 (Urban-724, Rural-399)	58 (Urban-39, rural-19)	5.16	Low Risk
20	Jha (2008) [31]	Leh	North	School	3–15	Urban	Yes	843	35	4.1	Low Risk
21	Sarma (2016) [32]	Guwahati	North East	School	6–16	Urban	Yes	400	77	19.25	Low Risk
22	Kalikivayi (1997) [33]	Hyderabad	South	School	3–18	Urban	Yes	3987	341	8.6	Low Risk
23	Kannan (2016) [34]	Chennai	South	School	6–12	Urban, Rural	Yes	1203 (Urban-603, Rural-600)	88 (Urban-52, rural-36)	7.3	Low Risk
24	Murthy (2014) [35]	Chittoor	South	School	5–16	Rural	Yes	1412	34	2.4	Moderate risk
25	Basu (2011) [36]	Surat	West	School	7–15	Urban	Yes	3002	418	13.9	Low Risk
26	Megala (2015) [37]	Krishnanagar	South	School	10–14	Urban	Yes	422	83	19.7	Low Risk
27	Meundi (2014) [38]	Kodagu	South	School	5–17	Rural	Yes	1938	332	17.13	Low Risk
28	Saha (2017) [39]	Kolkata	East	School	5–15	Urban	Yes	1840	151	8.2	Low Risk
29	Murthy (2002) [40]	Delhi	North	Population	5–15	Urban	Yes	5696	422	7.4	Low Risk
30	Krishnan (2015) [41]	Puducherry	South	School	9–14	Urban	Yes	1460	100	6.8	Moderate risk
31	Singh (2019) [42]	Gurugram	North	School	5–15	Urban	Yes	1234	261	21.1	Low Risk
32	Padhye (2009) [43]	Pune	West	School	5–15	Urban, Rural	Yes	12422 (Urban-5021, Rural-7401)	268 (Urban-160, rural-108)	2.15	Low Risk
33	Shukla (2018) [44]	Delhi	North	School	9–12	Urban	Yes	6056	152	2.5	Low Risk
34	Kumar (2014) [45]	Pune	West	School	6–16	Urban	NA	1157	68	5.9	Low Risk
35	Pavithra (2013) [46]	Bangalore	South	School	7–15	Urban, Rural	Yes	1378 (Urban-687, Rural-691)	61 (Urban-38, rural-23)	4.4	Low Risk
36	Singh (2015) [47]	Bhopal	Central	School	6–10	Urban, Rural	Yes	560 (Urban-280, Rural-280)	30 (Urban-16, rural-14)	5.35	Moderate risk
37	Cholera (2018) [48]	Pune	West	School	5–15	Urban	Yes	500	113	22.6	Moderate risk
38	Rahman (2015) [49]	Dibrugarh	North East	School	10–15	Urban	Yes	600	43	7.17	Low Risk
39	Kotabal (2017) [50]	Shivamogga	South	School	13–16	Urban	Yes	300	69	23	Moderate risk
40	Bigyabati (2016) [51]	Thoubal	North East	School	5–15	Rural	Yes	1770	108	6.1	Low Risk
41	Ravinder (2016) [52}	Warangal	South	School	7–12	Urban	Yes	5000	90	1.8	Moderate risk
42	Hashia (2017) [53]	Jammu	North	School	5–16	Rural	Yes	642	28	4.36	Low Risk
43	Saxena (2015) [8]	Delhi	North	School	5–15	Urban	Yes	9884	1297	13.12	Low Risk
44	Naik (2013) [54]	Ahmednagar	West	School	6–15	Rural	Yes	1095	45	4.1	Moderate risk
45	Samant (2015) [55]	Loni	West	School	10–12	Rural	Yes	1220	209	17.13	Moderate risk
46	Sandeep (2015) [56]	Hubli	South	School	7–15	Urban	Yes	2400	109	4.54	Moderate risk
47	Kumar K. (2016) [57]	Imphal	North East	School	11–13	Urban	Yes	302	88	29.14	Moderate risk
48	Sharma (2009) [58]	Rohtak	North	School	6–15	Rural	Yes	1265	153	12.7	Low Risk
49	Shakeel (2016) [59]	Dehradun	North	School	5–16	Urban	Yes	3146	156	5	Low Risk
50	Kumar (2016) [60]	Hyderabad	South	School	12–16	Urban	Yes	600	120	20	Low Risk
51	Sethi (2000) [61]	Ahmedabad	West	School	12–17	Urban	Yes	1647	265	16	Moderate risk
52	Tirkey (2018) [62]	Nalhar	Central	School	5–10	Urban	Yes	1300	128	9.84	Moderate risk
53	Uzma (2009) [63]	Hyderabad	South	School	7–15	Urban, Rural	Yes	3314 (Urban-1789, Rural-1525)	248 (Urban-229, rural-19)	7.48	Low Risk
54	Sharma (2018) [64]	Kangra	North	School	5–12	Urban	Yes	1007	33	3.27	Low Risk
55	Karavadi (2018) [65]	Bangalore	South	School	7–16	Rural	Yes	1140	48	4.21	Low Risk
56	Trivedi (2012) [66]	Sabarkantha	West	Population	7–15	Rural	Yes	452	18	4.1	Low Risk
57	Warad (2014) [67]	Devangere	South	School	10–12	Urban	Yes	7496	396	5.28	Low Risk
58	Warkad (2018) [68]	Bhubaneshwar	East	School	6–17	Urban	Yes	10038	56	0.63	Low Risk
59	Shukla (2016) [69]	Jabalpur	Central	School	5–15	Rural	Yes	200	4	2	Moderate risk

*NA- not available

** Based on Standard quality assessment tool given by Hoy et al. [10].

### Prevalence of myopia in 5–15 year age group

**Fig 1:**
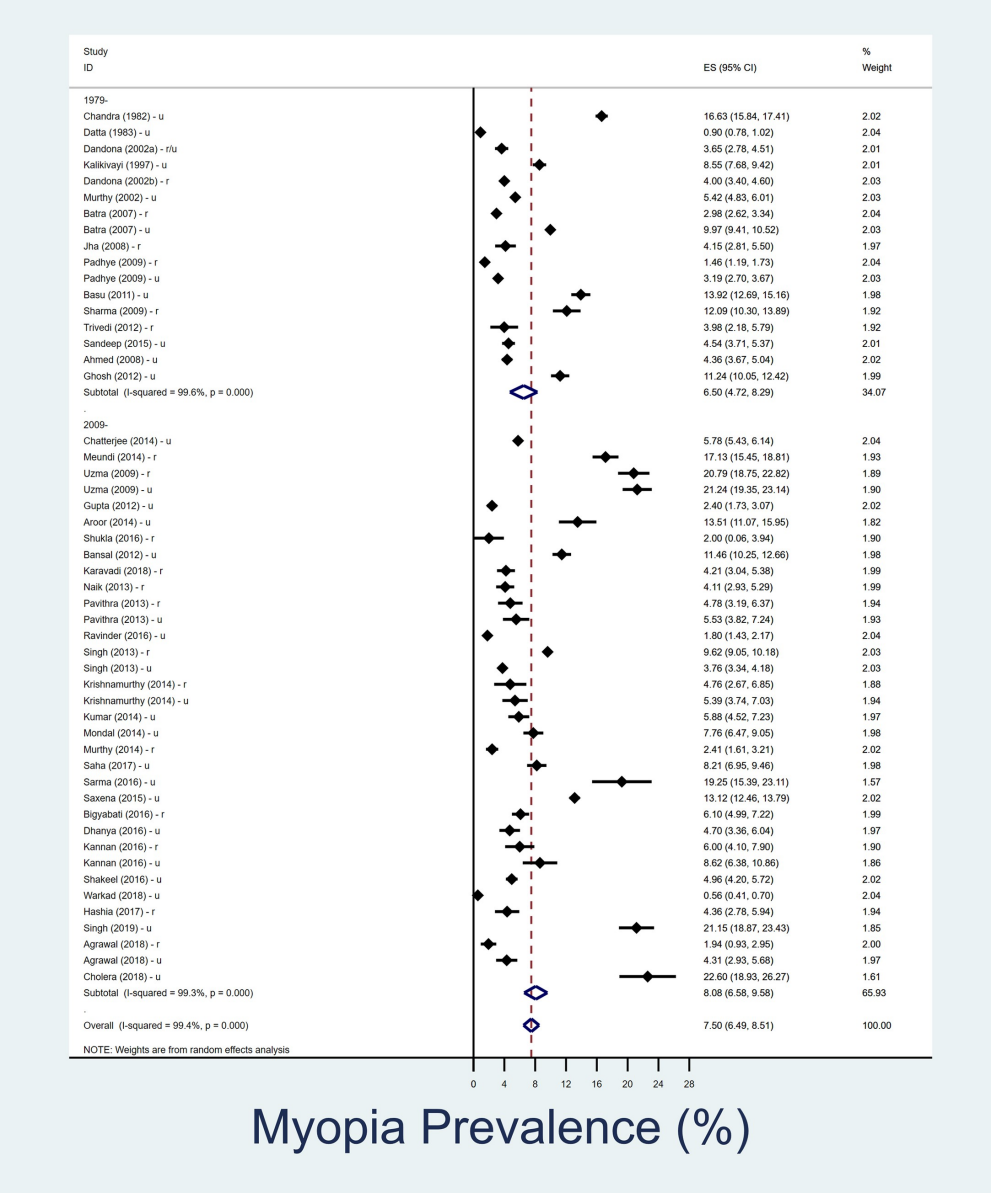
Forest plot showing overall prevalence of myopia in school going children (5–15 year) and its decadal variation. The datasets which represented urban and rural data are separately denoted as ‘u’ and ‘r’ respectively. Those studies in which urban/rural segregated data was not available are denoted as ‘r/u’.

**Fig 2:**
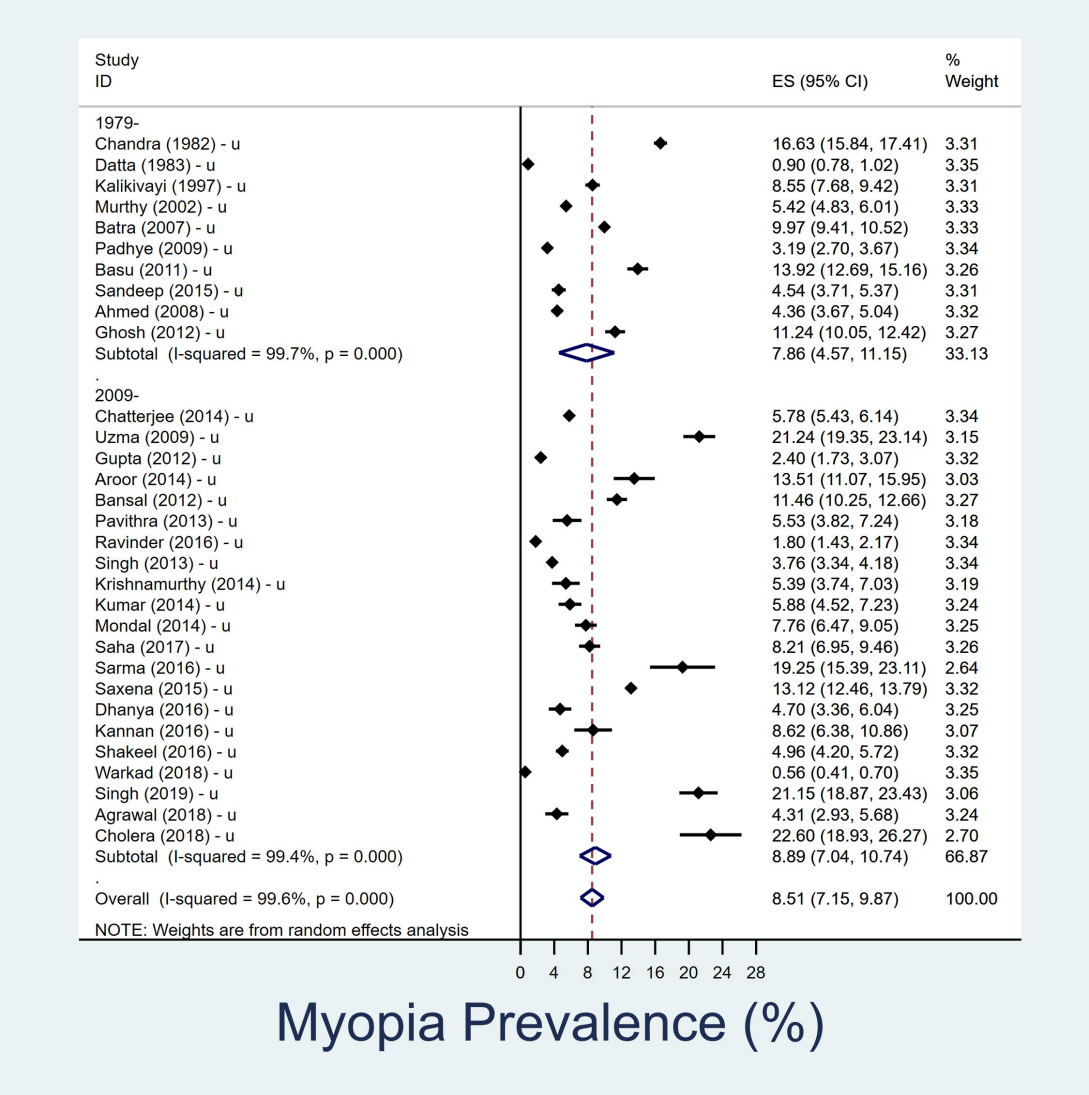
Forest plot showing prevalence of myopia in school going children (5–15 year) in urban setting and its decadal variation. The datasets which represented urban and rural data are separately denoted as ‘u’ and ‘r’ respectively. Those studies in which urban/rural segregated data was not available are denoted as ‘r/u’.

**Fig 3:**
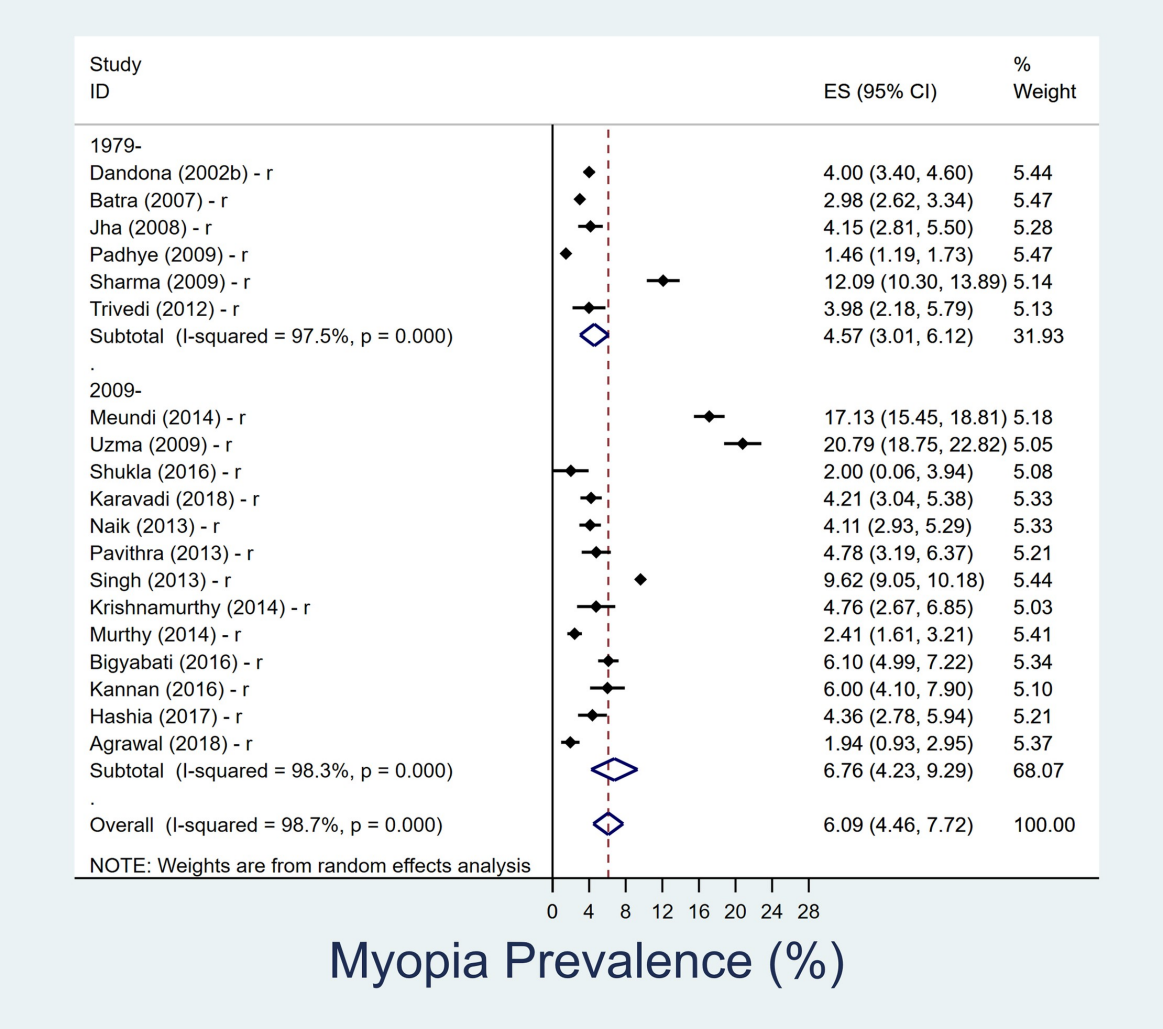
Forest plot showing prevalence of myopia in school going children (5–15 year) in rural setting and its decadal variation. The datasets which represented urban and rural data are separately denoted as ‘u’ and ‘r’ respectively. Those studies in which urban/rural segregated data was not available are denoted as ‘r/u’.

The summary of the results is shown in Table 2. In the 5-15-year age group, overall pooled prevalence of myopia over last four decades is 7.5% (95% CI, 6.5–8.5%) (Fig 1). The overall pooled prevalence of myopia in urban children is 8.5% (95% CI, 7.1–9.9%) and in rural settings is 6.1% (95% CI, 4.5–7.7%) in past four decades (Figs 2 and 3). In rural Indian children, the prevalence of myopia increased from 4.6% (95% CI, 3.0–6.1) in 1980–2008 to 6.8% (95% CI, 4.2–9.3) in 2009–2019. The increment among urban Indian children was lower, changing from 7.9% (95% CI, 4.6–11.2) in 1980–2008 to 8.9% (95% CI, 7.1–10.7) in 2009–2019. The heterogeneity of the studies included in pooled analysis, as well as urban-rural subgroups analysis were high (S2 and S3 Figs).

**Table 2:** Meta-analysis of prevalence of myopia in Indian school-age children, overall and stratified by time-periods and rural-urban population during 1980–2019.

	5–15 years age group		11–15 years age sub-group	
	Number of study datasets	Prevalence of Myopia (%) [95% CI]	Number of study datasets	Prevalence of Myopia (%) [95% CI]
**Rural and Urban**				
Overall	51	7.5 (6.5–8.5)	26	10.7 (9–12.4)
1980–2008 period	17	6.4 (4.7–8.1)	11	6.6 (4.8–8.3)
2009–2019 period	34	8.1 (6.6–9.6)	15	14.2 (11.2–17.2)
**Rural**				
Overall	19	6.1 (4.5–7.7)	7	10 (6.4–13.5)
1980–2008 period	6	4.6 (3.0–6.1)	3	6.9 (2.1–11.8)
2009–2019 period	13	6.8 (4.2–9.3)	4	12.3 (5.4–19.2)
**Urban**				
Overall	31	8.5 (7.1–9.9)	18	11.5 (9.3–13.6)
1980–2008 period	10	7.9 (4.6–11.2)	7	6.8 (4.1–9.4)
2009–2019 period	21	8.9 (7.1–10.7)	11	15.0 (11.2–18.7)

### Prevalence of myopia in 11–15 year age group

**Fig 4:**
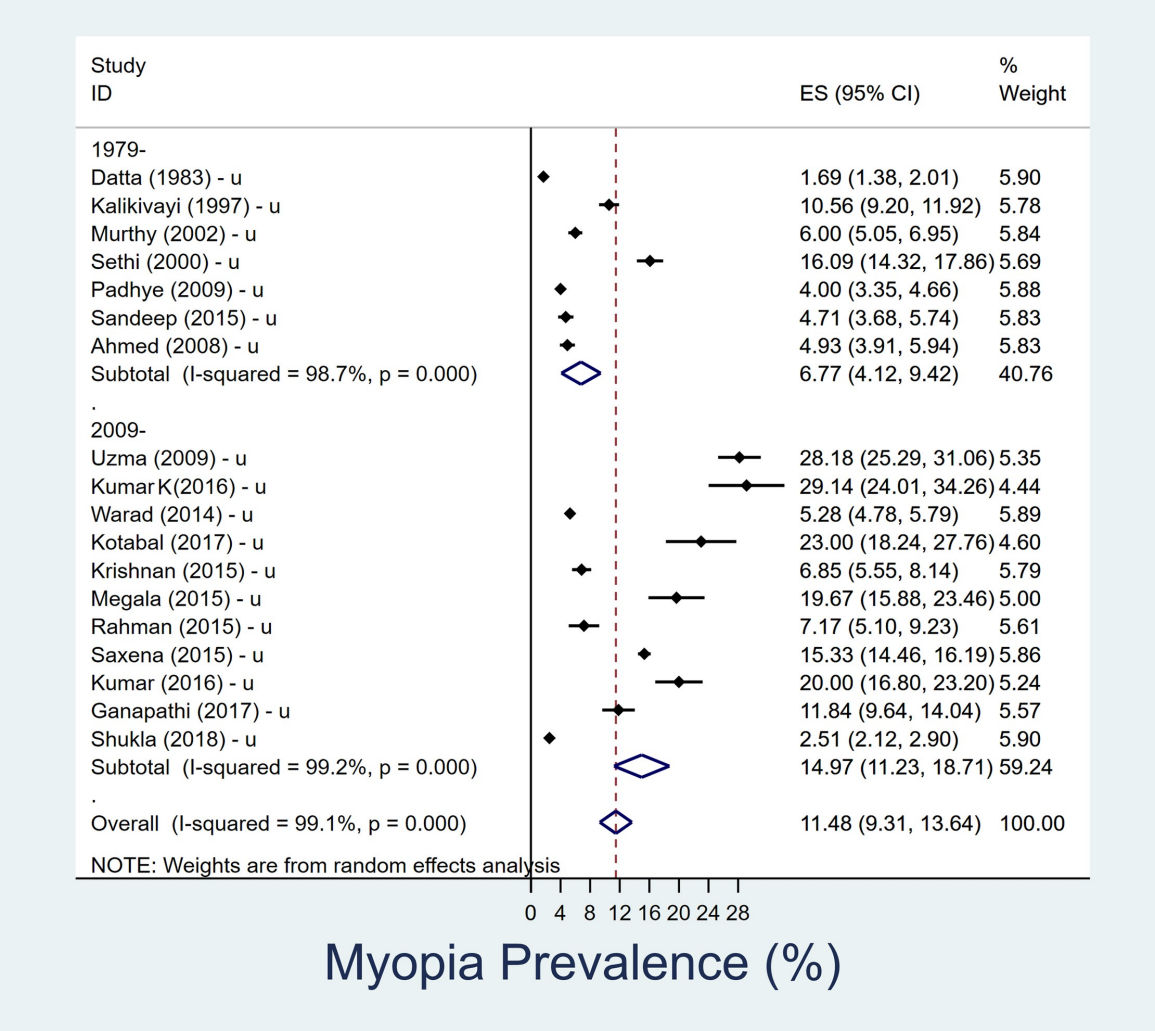
Forest plot showing change in prevalence of myopia over time in urban adolescent age group (11–15 years). The datasets which represented urban and rural data are separately denoted as ‘u’ and ‘r’ respectively. Those studies in which urban/rural segregated data was not available are denoted as ‘r/u’.

In the older 11-15-year age sub-group, the pooled prevalence of myopia over last four decades was 10.7% (95% CI, 9–12.4). Prevalence of myopia increased, in rural children aged 11–15 years, from 6.9% (95% CI, 2.1–11.8) in 1980–2008 to 12.3% (95% CI, 5.4–19.2) between 2009–2019 (Table 2). Similarly, near doubling of prevalence from 6.8% (95% CI, 4.1–9.4) in 1980–2008 to 15.0% (95% CI, 11.2–18.7) in 2009–2019 was observed among urban children aged 11–15 years (Fig 4). The heterogeneity of the pooled estimates was high.

### Sensitivity analysis

Eighteen studies classified as “high risk” were included in the sensitivity analysis (S2 Table). Similar trends were obtained as main analysis, with 7.4% (95% CI, 7–7.8%) overall pooled prevalence of myopia over last four decades in 5–15 year age group. The overall pooled prevalence of myopia in urban school going children (5–15 years age) was 9.2% (95% CI, 8.2–10.2%) and in rural settings was 7% (95% CI, 5.5–8.5%) in past four decades. Additional details of sensitivity analysis are available in S3 Table.

## Discussion

Myopia is emerging as a major public health problem worldwide [2, 5]. School going children are one of the most important risk group who constitute a large part of the Indian population [4, 5]. The current systematic review estimates the pooled prevalence of myopia, with a focus on studying rural-urban differences and time trends, and included fifty-nine quality assessed studies ensuring adequate rural-urban representation over different time intervals in the main analysis. Results show that the crude prevalence of myopia over last four decades is 7.5% in 5-15-year age group, being 8.5% and 6.1% in urban and rural school going children respectively. The prevalence has increased in rural India from 4.6% in 1980–2008 to 6.8% in 2009–2019, compared to a change from 7.9% to 8.9% in urban India during the same period.

Our results show that there is an increasing trend of myopia in India over the last four decades. Other meta-analyses from different parts of the world have also shown similar trends [2, 6, 88–91]. The prevalence of myopia is much less in Indian school going children as compared to other Asian countries where it could be as high as 70–80% [2, 88, 89]. While the prevalence may not be as high as that of East Asian countries, the actual numbers of myopes will be large considering our huge population and that 29% of the population consists of children less than 15 years of age (National Health Profile 2015, published by Government of India) [88–92]. This epidemiological variation also holds great importance as it pertains to world’s second most populated country which has more than 40% of young population who are at risk of developing myopia. Holden et al has estimated the prevalence of myopia in South Asia region (which includes India) to be around 20% in 2010, 38% in 2030 and 53% in 2050 [2]. We have found a lower prevalence of myopia in school going children in India over the last four decades as compared to other Asian countries where myopia is far more prevalent. Rudnicka et al has also found that increment in myopia prevalence in South Asian countries is less as compared to East Asian countries [90]. Thus, various meta-analyses which predicts global myopia trends fail to bring out this regional variation due to under representation of Indian studies [2, 6, 90].

This study has shown for the first time that there appears to be a significant rise in the prevalence of myopia in rural school going children. The percentage increment in myopia prevalence among rural school going children was 4 times more as compared to their urban counterparts, in the last decade (48% vs 12%). This is a novel epidemiological finding challenging the previous notion that myopia was less prevalent in rural areas in India as compared to urban areas [5, 91]. Systematic review by Sheeladevi et al. showed very low prevalence of myopia in rural settings as compared to urban settings in Indian children (3.5% vs 10.8%) [91]. While this might be a result of a demographic transition, their study assessed only eight school based and four population based studies.

There could be multiple reasons for the increase observed in rural school children. For the past few years, many Indian villages have become developed with access to basic amenities just like their urban counterparts. India is also witnessing a digital revolution starting from the past decade with increasing number of televisions, mobiles, laptops and computers. Internet usage has increased dramatically owing to reduced data tariff, low cost smartphones and improved telecom connectivity in Indian villages. This might have resulted in decreased outdoor activities, increased near work, and computer-related visual stress and fatigue [5, 90]. Changing schooling pattern to high pressure education system can also be another contributory factor [93, 94]. While direct causal relationship may be difficult to prove, but the rapidly changing environment (nurture) especially the ongoing urbanisation of rural environment in India could be implicated as a potential factor for this rising myopic prevalence.

This study confirms the findings of existing literature that urban adolescents (11–15 year age group) constitute an important ‘at risk’ subset of the general population requiring immediate attention and intervention where the prevalence of myopia increased to more than double in the last decade. Rural adolescents are also achieving the similar growth rate. Similar trend was obtained in other countries as well because myopia tends to develop after the natural curve of emmetropisation is over [3, 88, 89, 95].

India is geographically and demographically a large country with distinct regional identity and characteristics. Lack of studies reflecting the myopia prevalence from different regions of India and long-time gap between these studies were some important limitations of the study. The studies using inappropriate methodology, not published in English or where the relevant details in study text was unavailable, were excluded. We could not evaluate the prevalence and increment of high myopia which is important to prevent myopia related complications. Although numerous studies have shown an effect of gender on the myopia prevalence, gender-based variations could not be assessed due to limited data availability. Despite great heterogeneity in the results of the studies, we tried to address the differences and bring out some meaningful trends by using stratification, subgroup analysis and random effects model.

Similar trends were noted even after including eighteen high risk studies in the sensitivity analysis. Most of the studies which had poor methodological quality were conducted in the last decade. By excluding high risk studies, we adopted a conservative approach. The sensitivity analysis reaffirms the possibility of definite change in epidemiology of myopia in India over time. This large database is also one of the strengths of the present study which has helped to predict better trends and highlight subtle variations in epidemiology of myopia. Assessment of the time trends can be accurately done by observing the same population though repeated surveys at definite time intervals but these are rarely collected. Thus, the trend analysis from compiling available data might help in planning policies and setting priorities.

Myopia control programs require consistent efforts to increase awareness about risk factors, encourage lifestyle modification and changes in the school curriculum and education policy of the country. Therefore, this review should help stimulate the initiation of various preventive and corrective measures for myopia control, resource planning and infrastructure augmentation especially targeting the school going children [90, 95, 96].

## Conclusion

To conclude, this is the first Indian study to show and compare the prevalence of myopia in urban and rural settings over the last four decades. It has shown for the first time the rapidly rising trend of myopia in rural school going children compared to their urban counterparts. This should result in adoption of urgent preventive and curative measures among various stakeholders to tackle this menace on time. Future prospective studies should be planned among various diverse regions of India to elucidate the trend of myopia and study various local epidemiological risk factors involved.

## Supporting information

S1 Fig Summary of review strategy–PRISMA flow diagram. (TIF)

S2 Fig Funnel plot of the included studies which estimated prevalence of myopia in urban setting. (TIF)

S3 Fig Funnel plot of the included studies which estimated prevalence of myopia in rural setting. (TIF)

S1 Table Quality assessment of various eligible studies included in the sensitivity analysis. Standard quality assessment tool given by Hoy et al. Aggregate score <4- low risk, 4–6 moderate risk, >6 high risk. High risk studies are excluded (0- low risk, 1- high risk) [10].
(DOCX)

S2 Table Characteristics of various studies that were excluded from the final meta-analysis. *NA- not available, ** Based on Standard quality assessment tool given by Hoy et al. [10].
(DOCX)

S3 Table Summary of results of sensitivity analysis which included high risk studies (decadal variations and urban-rural variation). (DOCX)

S1 File Detailed search strategy. (DOCX)

S2 File PRISMA 2009 checklist. (DOC)

S3 File Supplementary data package. (ZIP)
