# Supplementary figures and images for: Prevalence of myopia in Indian school children: Meta-analysis of last four decades
Source: PLoS One. 2020 Oct 19;15(10):e0240750. doi: 10.1371/journal.pone.0240750 (PMC7571694; doi:10.1371/journal.pone.0240750)

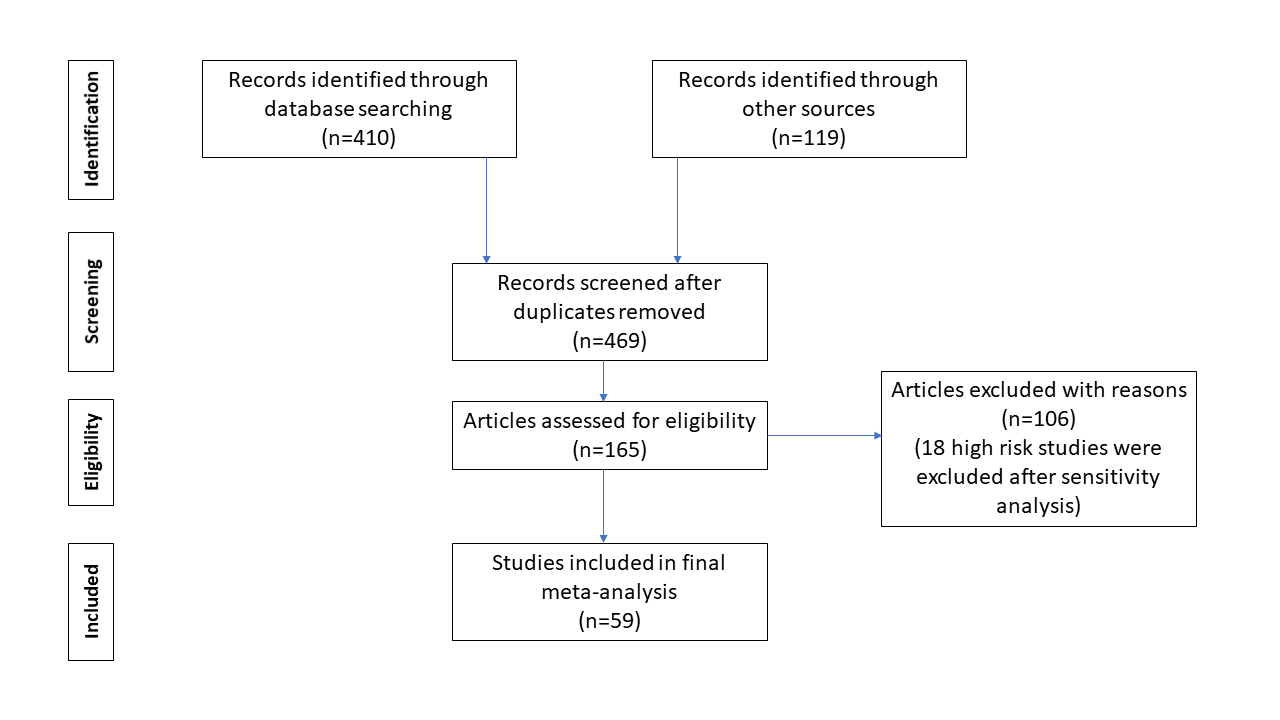

Supplement: S1 Fig — (TIF) [file pone.0240750.s001.tif]

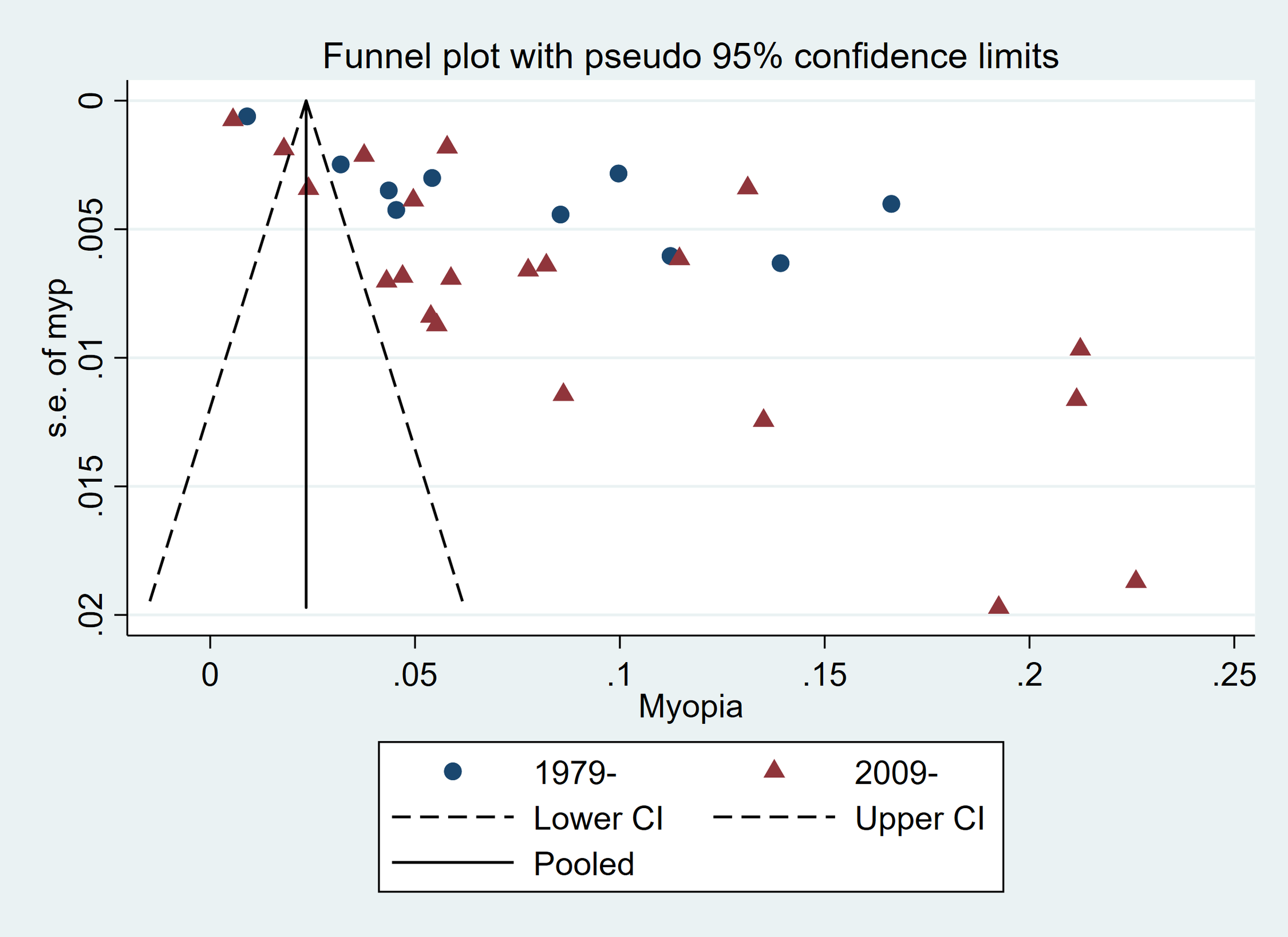

Supplement: S2 Fig — (TIF) [file pone.0240750.s002.tif]

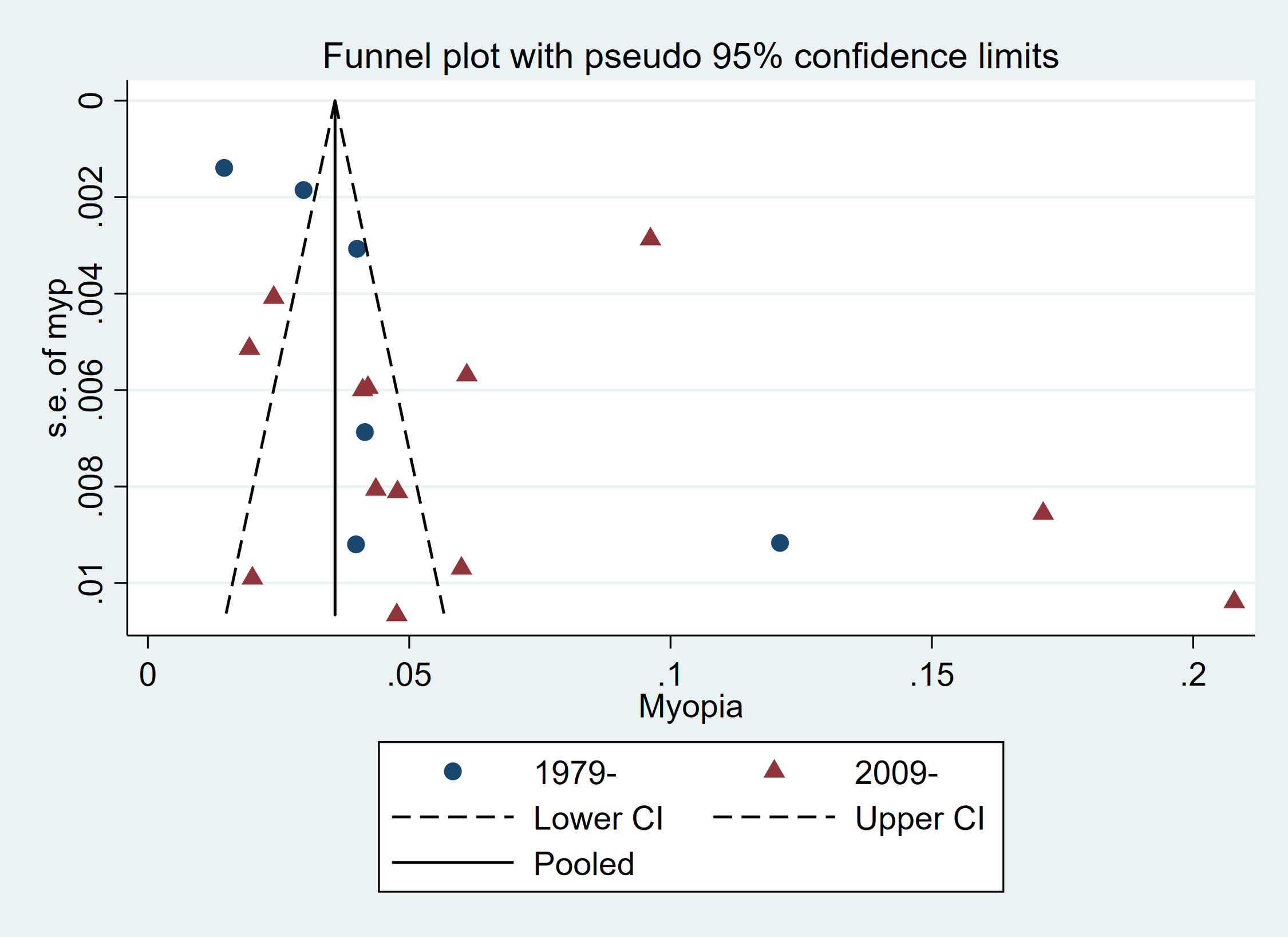

Supplement: S3 Fig — (TIF) [file pone.0240750.s003.tif]
