# Supplementary material for: Prevalence of myopia in Indian school children: Meta-analysis of last four decades
Source: PLoS One. 2020 Oct 19;15(10):e0240750. doi: 10.1371/journal.pone.0240750 (PMC7571694; doi:10.1371/journal.pone.0240750)
Supplement: S1 Table — Standard quality assessment tool given by Hoy et al. Aggregate score <4- low risk, 4–6 moderate risk, >6 high risk. High risk studies are excluded (0- low risk, 1- high risk) [10]. (DOCX) [file pone.0240750.s004.docx]

**S1 Table: Quality assessment of various eligible studies included in the sensitivity analysis.**

| **First Author (Year of Publication) [Citation]** | **1. Represent- ativeness of target population** | **2. Representat-iveness of sampling frame** | **3. Random selection** | **4. Non-response bias minimized (< 75% or MCAR)** | **5. Data collected from subject?** | **6. Case definition acceptable?** | **7. Measurement tools valid and reliable ?** | **8. Uniform assessment of all ?** | **9. Prevalence correctly calculated ?** | **Overall Assessment** |
| --- | --- | --- | --- | --- | --- | --- | --- | --- | --- | --- |
| Ahmed (2008) [12] | 0 | 0 | 1 | 1 | 0 | 0 | 0 | 0 | 0 | Low Risk |
| Bansal (2012) [13] | 0 | 1 | 1 | 1 | 0 | 0 | 1 | 0 | 0 | Moderate risk |
| Aroor (2014) [14] | 1 | o | o | 1 | 0 | 1 | 1 | 1 | 0 | Moderate risk |
| Ande (2015) [15] | 0 | 0 | 0 | 1 | 0 | 0 | 0 | 0 | 0 | Low Risk |
| Mondal (2014) [16] | 0 | 0 | 0 | 0 | 0 | 1 | 0 | 0 | 0 | Low Risk |
| Gupta (2012) [17] | 0 | 0 | 0 | 1 | 0 | 1 | 1 | 0 | 0 | Low Risk |
| Datta (1983) [18] | 0 | 1 | 1 | 1 | 0 | 0 | 1 | 1 | 0 | Moderate risk |
| Batra (2007) [19] | 0 | 0 | 1 | 1 | 0 | 1 | 0 | 0 | 0 | Low Risk |
| Chandra (1982) [20] | 0 | 1 | 1 | 1 | 0 | 1 | 0 | 1 | 0 | Moderate risk |
| Chatterjee (2014) [21] | 0 | 0 | 1 | 1 | 0 | 1 | 0 | 0 | 0 | Low Risk |
| Dandona (2002a) [22] | 0 | 0 | 0 | 0 | 0 | 0 | 0 | 0 | 0 | Low Risk |
| Dandona (2002b) [23] | 0 | 0 | 0 | 0 | 0 | 0 | 0 | 0 | 0 | Low Risk |
| Das (2007) [24] | 0 | 1 | 1 | 1 | 0 | 1 | 1 | 1 | 0 | Moderate risk |
| Agrawal (2018) [25] | 0 | 0 | 0 | 0 | 0 | 0 | 1 | 0 | 0 | Low Risk |
| Dhanya (2016) [26] | 0 | 1 | 1 | 1 | 0 | 0 | 0 | 1 | 0 | Moderate risk |
| Ganapathi (2017) [27] | 0 | 1 | 1 | 1 | 0 | 1 | 1 | 0 | 0 | Moderate risk |
| Ghosh (2012) [28] | 1 | 0 | 0 | 1 | 0 | 1 | 0 | 0 | 0 | Low Risk |
| Singh (2013) [29] | 1 | 1 | 1 | 1 | 0 | 1 | 0 | 0 | 0 | Moderate risk |
| Krishnamurthy (2014) [30] | 1 | 1 | 1 | 0 | 0 | 0 | 0 | 0 | 0 | Low Risk |
| Jha (2008) [31] | 0 | 0 | 1 | 0 | 0 | 0 | 0 | 1 | 0 | Low Risk |
| Sarma (2016) [32] | 0 | 0 | 0 | 1 | 0 | 0 | 0 | 0 | 0 | Low Risk |
| Kalikivayi (1997) [33] | 0 | 0 | 0 | 0 | 0 | 0 | 0 | 0 | 0 | Low Risk |
| Kannan (2016) [34] | 0 | 0 | 0 | 0 | 0 | 1 | 0 | 0 | 0 | Low Risk |
| Murthy (2014) [35] | 0 | 1 | 1 | 1 | 0 | 1 | 1 | 1 | 0 | Moderate risk |
| Basu (2011) [36] | 0 | 0 | 0 | 0 | 0 | 1 | 0 | 0 | 0 | Low Risk |
| Megala (2015) [37] | 0 | 0 | 0 | 1 | 0 | 1 | 1 | 0 | 0 | Low Risk |
| Meundi (2014) [38] | 0 | 0 | 1 | 0 | 0 | 1 | 0 | 0 | 0 | Low Risk |
| Saha (2017) [39] | 0 | 0 | 0 | 1 | 0 | 0 | 0 | 0 | 0 | Low Risk |
| Murthy (2002) [40] | 0 | 0 | 0 | 0 | 0 | 0 | 0 | 0 | 0 | Low Risk |
| Krishnan (2015) [41] | 0 | 1 | 1 | 1 | 0 | 0 | 1 | 0 | 0 | Moderate risk |
| Singh (2019) [42] | 0 | 0 | 1 | 0 | 0 | 0 | 0 | 0 | 0 | Low Risk |
| Padhye (2009) [43] | 0 | 0 | 0 | 1 | 0 | 0 | 0 | 0 | 0 | Low Risk |
| Shukla (2018) [44] | 0 | 0 | 0 | 1 | 0 | 0 | 0 | 0 | 0 | Low Risk |
| Kumar (2014) [45] | 0 | 0 | 0 | 1 | 0 | 1 | 1 | 0 | 0 | Low Risk |
| Pavithra (2013) [46] | 0 | 1 | 1 | 1 | 0 | 0 | 0 | 0 | 0 | Low Risk |
| Singh (2015) [47] | 0 | 1 | 1 | 1 | 0 | 1 | 0 | 1 | 0 | Moderate risk |
| Cholera (2018) [48] | 1 | 1 | 1 | 0 | 0 | 1 | 1 | 1 | 0 | Moderate risk |
| Rahman (2015) [49] | 0 | 0 | 0 | 1 | 0 | 0 | 0 | 1 | 0 | Low Risk |
| Kotabal (2017) [50] | 1 | 1 | 1 | 1 | 0 | 1 | 0 | 0 | 0 | Moderate risk |
| Bigyabati (2016) [51] | 0 | 0 | 0 | 0 | 0 | 1 | 1 | 0 | 0 | Low Risk |
| Ravinder (2016) [52} | 1 | 1 | 1 | 0 | 0 | 1 | 1 | 1 | 0 | Moderate risk |
| Hashia (2017) [53] | 0 | 0 | 1 | 1 | 0 | 1 | 0 | 0 | 0 | Low Risk |
| Saxena (2015) [10] | 0 | 0 | 0 | 0 | 0 | 0 | 0 | 0 | 0 | Low Risk |
| Naik (2013) [54] | 0 | 1 | 1 | 0 | 0 | 1 | 0 | 1 | 0 | Moderate risk |
| Samant (2015) [55] | 0 | 1 | 1 | 1 | 0 | 1 | 1 | 1 | 0 | Moderate risk |
| Sandeep (2015) [56] | 0 | 0 | 1 | 1 | 0 | 1 | 1 | 1 | 0 | Moderate risk |
| Kumar K. (2016) [57] | 0 | 0 | 0 | 1 | 0 | 1 | 1 | 1 | 0 | Moderate risk |
| Sharma (2009) [58] | 0 | 0 | 0 | 1 | 0 | 1 | 1 | 0 | 0 | Low Risk |
| Shakeel (2016) [59] | 1 | 0 | 1 | 1 | 0 | 0 | 0 | 0 | 0 | Low Risk |
| Kumar (2016) [60] | 0 | 0 | 0 | 1 | 0 | 1 | 0 | 0 | 0 | Low Risk |
| Sethi (2000) [61] | 1 | 0 | 0 | 0 | 0 | 1 | 1 | 1 | 0 | Moderate risk |
| Tirkey (2018) [62] | 1 | 0 | 1 | 1 | 0 | 1 | 0 | 0 | 0 | Moderate risk |
| Uzma (2009) [63] | 0 | 1 | 1 | 1 | 0 | 0 | 0 | 0 | 0 | Low Risk |
| Sharma (2018) [64] | 0 | 0 | 0 | 1 | 0 | 1 | 0 | 0 | 0 | Low Risk |
| Karavadi (2018) [65] | 0 | 0 | 0 | 1 | 0 | 0 | 0 | 0 | 0 | Low Risk |
| Trivedi (2012) [66] | 0 | 0 | 0 | 1 | 0 | 0 | 1 | 1 | 0 | Low Risk |
| Warad (2014) [67] | 0 | 1 | 1 | 1 | 0 | 0 | 0 | 0 | 0 | Low Risk |
| Warkad (2018) [68] | 1 | 0 | 0 | 0 | 0 | 0 | 0 | 0 | 0 | Low Risk |
| Shukla (2016) [69] | 1 | 1 | 1 | 1 | 0 | 1 | 1 | 0 | 0 | Moderate risk |
| Afroz (2005) [70] | 1 | 1 | 1 | 1 | 0 | 1 | 1 | 1 | 0 | High Risk |
| Ansari (2015) [71] | 1 | 1 | 1 | 1 | 0 | 1 | 1 | 1 | 1 | High Risk |
| Avinash (2019) [72] | 1 | 0 | 1 | 1 | 0 | 1 | 1 | 1 | 1 | High Risk |
| Bhat (2015) [73] | 1 | 1 | 1 | 1 | 0 | 1 | 1 | 1 | 1 | High Risk |
| Binu (2016) [74] | 1 | 1 | 1 | 1 | 0 | 1 | 1 | 1 | 0 | High Risk |
| Gupta (2011) [75] | 1 | 1 | 1 | 1 | 0 | 1 | 1 | 1 | 1 | High Risk |
| Hittalamani (2015) [76] | 1 | 1 | 1 | 1 | 0 | 0 | 1 | 1 | 1 | High Risk |
| John (2017) [77] | 1 | 1 | 1 | 1 | 0 | 1 | 1 | 1 | 1 | High Risk |
| Kavitha (2016) [78] | 1 | 1 | 1 | 1 | 0 | 1 | 1 | 1 | 0 | High Risk |
| Kemmanu (2016) [79] | 1 | 1 | 1 | 1 | 0 | 1 | 0 | 1 | 1 | High Risk |
| Kher (2017) [80] | 1 | 1 | 1 | 1 | 0 | 0 | 1 | 1 | 1 | High Risk |
| Maheshgauri (2016) [81] | 1 | 1 | 1 | 1 | 0 | 1 | 1 | 1 | 1 | High Risk |
| Pradhan (2018) [82] | 1 | 1 | 1 | 1 | 0 | 1 | 1 | 1 | 1 | High Risk |
| Rajendran (2014) [83] | 1 | 1 | 1 | 1 | 0 | 1 | 1 | 1 | 1 | High Risk |
| Rao (2016) [84] | 1 | 1 | 1 | 1 | 0 | 0 | 1 | 1 | 1 | High Risk |
| Sathyan (2018) [85] | 1 | 1 | 1 | 1 | 0 | 1 | 1 | 1 | 1 | High Risk |
| Shekhar (2019) [86] | 1 | 1 | 1 | 1 | 0 | 1 | 1 | 1 | 1 | High Risk |
| Sudhan (2009) [87] | 1 | 1 | 1 | 1 | 0 | 1 | 1 | 1 | 1 | High Risk |

*Standard quality assessment tool given by Hoy et al. Aggregate score <4- low risk, 4-6 moderate risk, >6 high risk. High risk studies are excluded. (0- low risk, 1- high risk) (10)
