# Supplementary material for: Prevalence of myopia in Indian school children: Meta-analysis of last four decades
Source: PLoS One. 2020 Oct 19;15(10):e0240750. doi: 10.1371/journal.pone.0240750 (PMC7571694; doi:10.1371/journal.pone.0240750)
Supplement: S2 Table — *NA- not available, ** Based on Standard quality assessment tool given by Hoy et al. [10]. (DOCX) [file pone.0240750.s005.docx]

| **S No.** | **First Author (Year of Publication)** | **Study Place** | **Region (India)** | **Coverage** | **Age Group**  **(years)** | **Coverage** | **Cycloplegic Refraction** | **Total Sample Size (Subdivision when both urban and rural data separately available)** | **No. of myopic cases (Subdivision when both urban and rural data available)** | **Prevalence of myopia (%)** | **Overall Quality Assessment Score**** |
| --- | --- | --- | --- | --- | --- | --- | --- | --- | --- | --- | --- |
| 1 | Afroz (2005) [70] | Srinagar | North | School | 6-16 | Urban, Rural | Yes | 1062 | 214 | 20.15 | High Risk |
| 2 | Ansari (2015) [71] | Aligarh | Central | School | 5-16 | Urban | Yes | 3127 | 45 | 1.43 | High Risk |
| 3 | Avinash (2019) [72] | Udupi | South | School | 5-15 | Urban, Rural | NA* | 1782 | 71 | 3.98 | High Risk |
| 4 | Bhat (2015) [73] | Srinagar | North | School | 10-16 | Urban | Yes | 720 | 51 | 7.08 | High Risk |
| 5 | Binu (2016) [74] | Kollam | South | School | 10-15 | Rural | Yes | 300 | 132 | 44 | High Risk |
| 6 | Gupta (2011) [75] | Hathras | Central | School | 3-12 | Rural | yes | 110 | 2 | 1.81 | High Risk |
| 7 | Hittalamani (2015) [76] | Hubli | South | School | 7-15 | Urban, Rural | Yes | 4429 | 266 | 6.0 | High Risk |
| 8 | John (2017) [77] | Vellore | South | School | 11-14 | Rural | Yes | 4138 | 18 | 0.43 | High Risk |
| 9 | Kavitha (2016) [78] | Theni | South | School | 5-15 | Urban | Yes | 1000 | 422 | 42.2 | High Risk |
| 10 | Kemmanu (2016) [79] | Tumkur | South | School | 0-15 | Rural | Yes | 23087 | 107 | 0.46 | High Risk |
| 11 | Kher (2017) [80] | Wardha | West | School | 5-16 | Rural | Yes | 500 | 198 | 39.6 | High Risk |
| 12 | Maheshgauri (2016) [81] | Pune | West | School | 3-13 | Urban | NA | 560 | 166 | 29.64 | High Risk |
| 13 | Pradhan (2018) [82] | Mullana | North | School | 6-12 | Urban, Rural | Yes | 1000 | 44 | 4.4 | High Risk |
| 14 | Rajendran (2014) [83] | Kollam | South | School | 10-12 | Rural | NA | 68 | 35 | 51.5 | High Risk |
| 15 | Rao (2016) [84] | Nizamabad | South | School | 4-15 | Urban, Rural | Yes | 531121 | 7414 | 1.4 | High Risk |
| 16 | Sathyan (2018) [85] | Thrissur | South | School | 6-17 | Urban | NA | 91628 | 149 | 0.16 | High Risk |
| 17 | Shekhar (2019) [86] | Goa | West | School | 6-10 | Urban, Rural | Yes | 817 | 49 | 5.99 | High Risk |
| 18 | Sudhan (2009) [87] | Satna | Central | School | 9-17 | Rural | NA | 68833 | 410 | 0.6 | High Risk |

**S2 Table: Characteristics of various studies that were excluded from the final meta-analysis.**

*NA- not available

** Based on Standard quality assessment tool given by Hoy et al. (10)
