# Supplementary material for: Prevalence of myopia in Indian school children: Meta-analysis of last four decades
Source: PLoS One. 2020 Oct 19;15(10):e0240750. doi: 10.1371/journal.pone.0240750 (PMC7571694; doi:10.1371/journal.pone.0240750)
Supplement: S3 Table — (DOCX) [file pone.0240750.s006.docx]

**S3 Table. Summary of results of sensitivity analysis which included high risk studies (decadal variations and urban-rural variation)**

|  | **5-15 years age group** | | **11-15 years age subgroup** | |
| --- | --- | --- | --- | --- |
|  | Number of study datasets | Prevalence of Myopia (%) [95% CI] | Number of study datasets | Prevalence of Myopia (%) [95% CI] |
| **Rural and Urban** |  |  |  |  |
| Overall | 62 | 7.4 (7- 7.8) | 30 | 11.5 (9.9- 13) |
| 1980-2008 period | 19 | 6.8 (5.5- 8.1) | 11 | 6.6 (4.8- 8.3) |
| 2009-2019 period | 43 | 7.7 (7.2- 8.2) | 19 | 15.3 (12.7- 17.9) |
| **Rural** |  |  |  |  |
| Overall | 21 | 7 (5.5- 8.5) | 10 | 12.7 (10.1- 15.2) |
| 1980-2008 period | 7 | 3.4 (2.6- 5.2) | 3 | 6.9 (2.1- 11.8) |
| 2009-2019 period | 14 | 8.8 (6- 11.6) | 7 | 18.1 (12.8- 23.3) |
| **Urban** |  |  |  |  |
| Overall | 35 | 9.2 (8.2- 10.2) | 19 | 11.2 (9.1- 13.3) |
| 1980-2008 period | 10 | 7.9 (4.6- 11.2) | 7 | 6.8 (4.1- 9.4) |
| 2009-2019 period | 25 | 9.8 (8.6- 11.1) | 12 | 14.3 (10.8- 17.8) |
