# Supplementary material for: Prevalence of myopia in Indian school children: Meta-analysis of last four decades
Source: PLoS One. 2020 Oct 19;15(10):e0240750. doi: 10.1371/journal.pone.0240750 (PMC7571694; doi:10.1371/journal.pone.0240750)
Supplement: S1 File — (DOCX) [file pone.0240750.s007.docx]

**MEDLINE and EMBASE databases**

**Textwords**

(((Short sight) OR (Myopia) OR (Myope) OR (Refractive error) OR (Ocular Refraction)).tw)

AND (((Incident) OR (Incidence) OR (Prevalence) OR (Population) OR (Survey)).tw)

AND (((Child) OR (Childhood) OR (Children) OR (Adolescent) OR (Adolescence) OR

(Teenage)).tw)

AND (((India) OR (Indian state) OR (Indian district) OR (Indian city) OR (Delhi) OR (Andhra Pradesh) OR (Assam) OR (Arunachal Pradesh) OR (Bihar) OR (Chattisgarh) OR (Goa) OR (Gujarat) OR (Haryana) OR (Himachal Pradesh) OR (Jharkhand) OR (Karnataka) OR (Kerala) OR (Madhya Pradesh) OR (Maharashtra) OR (Manipur) OR (Meghalaya) OR (Mizoram) OR (Nagaland) OR (Odisha) OR (Punjab) OR (Rajasthan) OR (Sikkim) OR (Tamil Nadu) OR (Telangana) OR (Tripura) OR (Uttar Pradesh) OR (Uttarakhand) OR (West Bengal) OR (Indian union territory)).tw)

**MESH headings (Medline)**

(Myopia/) OR (Refraction, ocular/) OR (Refractive errors/)

AND ((Incidence/) OR (Prevalence/) OR (Population/))

AND ((CHILD/) OR (ADOLESCENT/))

**Subject headings (Embase)**

((Myopia/) OR (High myopia/) OR (Refractive error/))

AND ((Incidence/) OR (Prevalence/) OR (Population/) OR (Health survey/))

AND ((CHILD/) OR (ADOLESCENT/))

Combine Textword, MESH and Subject headings search within Medline and Embase

**Web of Science database**

Topic Search TS= ((Myopia) OR (Myopic) OR (Short sight) OR (Refractive error) OR (Ocular

refraction))

AND ((Incident) OR (Incidence) OR (Prevalence) OR (Population) OR (Survey)

AND ((Child) OR (Childhood) OR (Children) OR (Adolescent) OR (Adolescence) OR (Teenage))

AND ((India) OR (Indian state) OR (Indian district))
